# Patient-Centered Summarization Framework for AI Clinical Summarization: Mixed Methods Study

**DOI:** 10.2196/87061

**Published:** 2026-09-03

**Authors:** Maria Lizarazo Jimenez, Ana Gabriela Claros, Kieran Green, David Toro-Tobon, Felipe Larios, Sheena Asthana, Camila Wenczenovicz, Kerly Guevara Maldonado, Luis Vilatuna-Andrango, Ana Cristina Proano, Satya Sai Sri Bandi, Shubhangi Bagewadi, Megan E Branda, Misk Al Zahidy, Saturnino Luz, Mirella Lapata, Juan P Brito, Oscar J Ponce-Ponte

**Affiliations:** 1 Department of Medicine, Division of Endocrinology, Diabetes, Metabolism and Nutrition, Knowledge and Evaluation Research Unit, Care and AI Laboratory, Mayo Clinic, 100 2nd St SW, Rochester, MN, 55905, United States, 1 (507) 284-2511; 2 Division of Endocrinology, Diabetes, Metabolism, and Nutrition, Mayo Clinic, Rochester, MN, United States; 3 Centre for Health Technology, University of Plymouth, Plymouth, United Kingdom; 4 Division of Clinical Trials and Biostatistics, Department of Quantitative Health Sciences, Mayo Clinic, Rochester, MN, United States; 5 Usher Institute, University of Edinburgh, Edinburgh, Scotland, United Kingdom; 6 Derriford Hospital, University Hospitals Plymouth NHS Trust, Plymouth, England, United Kingdom

**Keywords:** patient-centered, large language models, LLMs, qualitative research, mixed methods, artificial intelligence

## Abstract

**Background:**

Large language models (LLMs) are increasingly demonstrating the potential to reach human-level performance in generating clinical summaries from patient-clinician conversations. LLMs are usually evaluated against clinical summaries that focus mainly on patients’ biology and not on their biography (eg, preferences, values, wishes, and concerns). To achieve patient-centered care, artificial intelligence clinical summarization must incorporate patient-centered domains, implemented through patient-centered summaries (PCSs).

**Objective:**

This study aimed to develop a framework to generate PCS that capture patients’ values, preferences, and wishes while ensuring clinical utility for clinicians, and assess if current open-source LLMs can achieve human-level performance in generating PCS.

**Methods:**

We developed a 4-step mixed methods process to define and evaluate PCS. First, 2 patient and public involvement and engagement groups were convened in the United Kingdom (10 patients and 8 clinicians), who participated in semistructured interviews exploring what personal and contextual information should be included in clinical summaries and how it should be structured for clinical use. Second, findings were translated into an annotation guideline, which was used by 8 clinician annotators to generate gold standard PCS from 88 transcribed patient-clinician consultations about the management of atrial fibrillation. Third, 16 consultations were used to iteratively develop and refine a prompt aligned with the annotation guideline. Finally, 5 LLMs (Llama-3.2-3B [Meta AI], Llama-3.1-8B [Meta AI], Mistral-8B [Mistral AI], Gemma-3-4B [Google DeepMind], and Qwen3-8B [Alibaba]) generated summaries from 72 consultations using zero-shot and few-shot prompting, which were evaluated against gold standard PCS using ROUGE-L (Recall-Oriented Understudy for Gisting Evaluation–Longest Common Subsequence) and BERTScore (Bidirectional Encoder Representations from Transformers Score) and assessed for correctness, completeness, conciseness, patient-centeredness, and fluency.

**Results:**

Patients emphasized that summaries should include (1) lifestyle routines and daily functioning as indicators of independence or disruption; (2) the presence and role of social support systems, especially during crises; (3) recent life events or stressors, such as trauma, loss, or caregiving demands; and (4) care preferences, values, and communication styles that provide meaning or reflect autonomy. Clinicians sought summaries that included a concise functional baseline, psychosocial context, and emotional cues, preferably in a structured, clinically digestible format. In the 72 consultations (mean age 70, SD 11 y; 32/72, 44.4% female), the best zero-shot performance was observed with Mistral-8B (ROUGE-L 0.189) and Llama-3.1-8B (BERTScore 0.673). The best few-shot prompting was found with 3 examples using Llama-3.1-8B (ROUGE-L 0.206 and BERTScore 0.683).

**Conclusions:**

The open-source LLMs we evaluated did not achieve human-level performance in generating PCSs. Without task-specific fine-tuning, current open-source LLMs cannot reach human-level performance in this task. Our framework serves as an innovative guideline for developing gold standard PCS for artificial intelligence clinical tasks.

## Introduction

Clinical summarization is an essential component of modern health care, as it allows clinicians to efficiently aggregate, organize, and synthesize complex patient data into concise, actionable insights that support clinical reasoning, decision-making, and care coordination [1]. High-quality summaries help reduce information overload, promote safer care transitions, and improve communication among providers and with patients [2-6]. Yet, preparing these summaries is time-consuming and contributes to documentation burden, which has motivated the development of artificial intelligence (AI) systems to automate the task [7-9]. These systems use natural language processing to generate concise summaries from electronic health records (EHRs) and patient-clinician encounters [10,11]. Recent work in clinical dialogue summarization has implemented multistage pipelines and fine-tuned large language models (LLMs) to improve the accuracy and coherence of patient-clinician summaries, reaching almost human-level performance [11-13].

However, current AI models are trained on clinical notes that have increasingly evolved to capture biological complexity as well as provide enough documentation to justify billing and reimbursement [14-16]. This dual purpose has shaped current summaries and the datasets used to guide current AI scribes, making them biologically centered and prioritizing pathophysiological details over patient preferences, their context, and what truly matters to them [13]. As a result, these models often produce clinical summaries that focus on medical issues and the clinician’s perspective, without fully incorporating patients’ values, preferences, and concerns [17,18].

Different forms of clinical summarization have demonstrated how patient-centered information can be integrated into routine care while having an impact on care outcomes [19-21]. For instance, including both patients and clinicians’ perspectives on after-visit summaries provides actionable instructions, medication explanations, and tailored self-management guidance that support patient understanding and engagement [19]. In rheumatology, written consultation summaries cocreated with patients support continuity of care and improve health literacy [20]. Similarly, tools such as surveys or questionnaires designed to elicit patient “values” have been transformed into narrative summary reports to communicate patients’ values to health care professionals and to facilitate values-based discussions and shared decision-making (SDM) for chronic conditions, improving alignment between care plans and patient preferences and reducing decisional conflict [21]. Despite these examples, there is currently no structured framework that defines how to systematically generate and evaluate patient-centered summaries (PCSs) that capture patients’ values and preferences while preserving clinical utility [22,23]. Additionally, the ability of current open-source LLMs to generate PCS has not yet been evaluated [13].

We conceptualize patient-centered summarization as a distinct subtype of AI clinical summarization, particularly relevant for patients with multimorbidity whose care decisions depend on goals, preferences, and lived context rather than condition-specific biomedical information alone [24]. Unlike traditional clinical summarization, which prioritizes biomedical extraction for billing and documentation [15,16,25], PCS emphasizes preferences, values, wishes, and concerns to support longitudinal decision-making and SDM [21,23]. This subtype is characterized by the need to capture both biomedical and patient-centered information, guided by structured patient-centered domains and requiring evaluation beyond conventional summarization metrics.

To ensure that PCS capture what matters to patients while remaining clinically useful, their definition must be context-dependent and informed by both patient and clinician perspectives through iterative feedback, enabling the development of adaptable, evolving gold standards for local benchmarking or model refinement [26,27]. Because this process can be time- and resource-intensive, we propose a patient and public involvement and engagement (PPIE)–driven approach and demonstrate the feasibility of this methodology [27-29]. Specifically, we (1) introduce a novel mixed methods framework for developing a context-aware, patient-centered, gold standard for clinical summarization, and (2) benchmark current open-source LLMs against gold standard PCS to establish a baseline for future improvement in this area.

## Methods

### Overview

**Figure 1.**
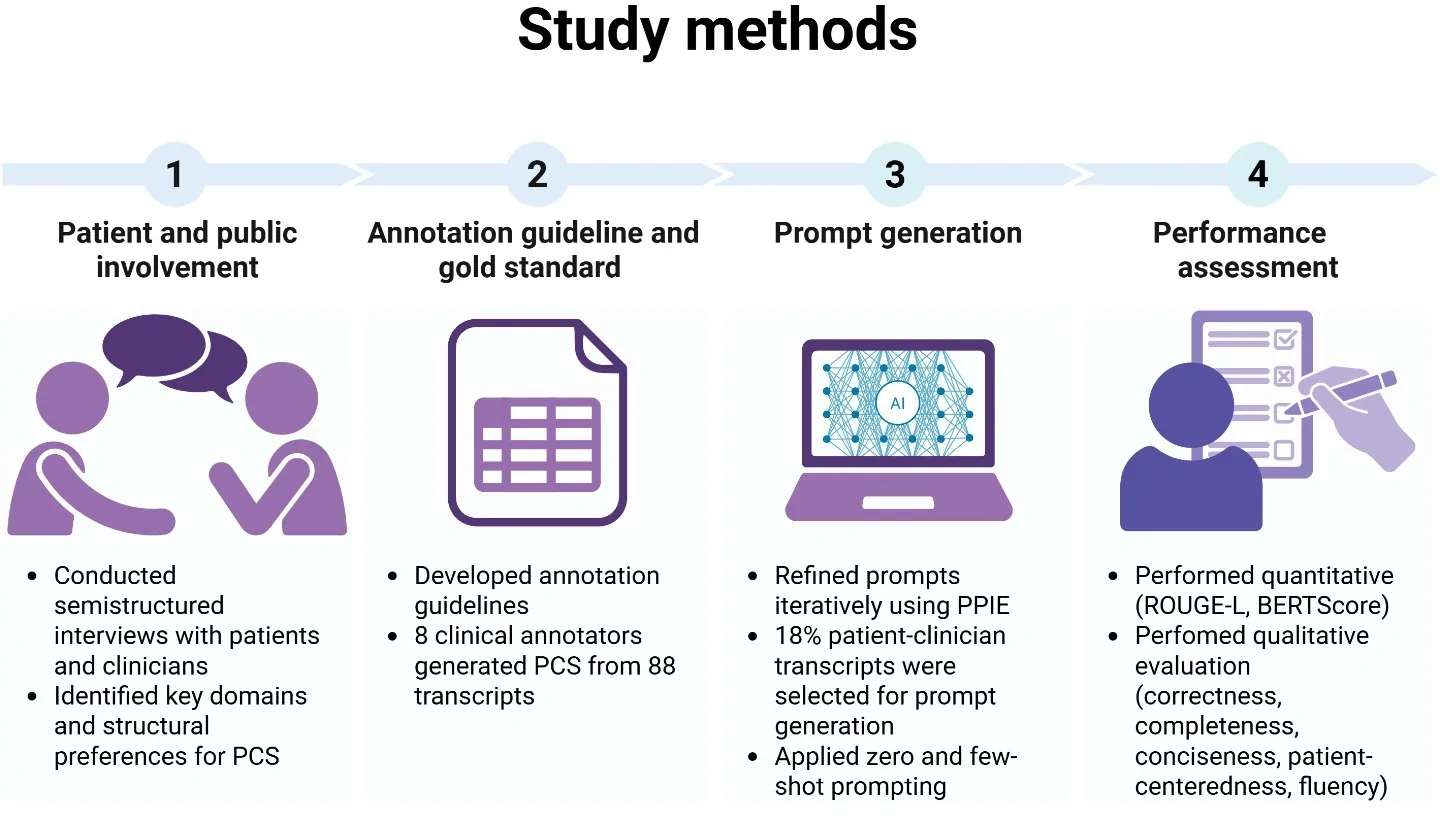
Overview of the 4-step study workflow for developing and evaluating patient-centered summaries (PCSs). The process included (1) patient and public involvement and engagement to define PCS domains, (2) development of annotation guidelines and creation of 88 gold standard PCSs, (3) prompt refinement using 16 consultations, and (4) benchmarking 5 open-source large language models on 72 held-out consultations using ROUGE-L (Recall-Oriented Understudy for Gisting Evaluation–Longest Common Subsequence), BERTScore (Bidirectional Encoder Representations from Transformers Score), and qualitative human evaluation. PCS: patient-centered summary; PPIE: patient and public involvement and engagement.

The framework to define, generate, and evaluate PCS followed a 4-step mixed methods design. First, we captured patients’ and clinicians’ perspectives into PCS by leveraging the PPIE methodology; 2 PPIE groups were convened to define the core components of a PCS through semistructured interviews. Second, these findings were translated into an annotation guideline, which 8 clinician annotators used to create gold standard PCS for 88 transcribed patient-clinician consultations. Third, a prompt was iteratively developed using 16 consultations and then used to generate PCS from 5 general-purpose, open-source LLMs (Llama-3.2-3B [Meta AI], Llama-3.1-8B [Meta AI], Mistral-8B, Gemma-3-4B [Google DeepMind], and Qwen3-8B [Alibaba Cloud]) with zero-shot and few-shot prompting techniques. Notably, none of these models were pretrained on clinical summaries from EHRs. Finally, the AI-generated summaries from the remaining 72 consultations were evaluated against the gold standard PCS using a quantitative analysis (ROUGE-L [Recall-Oriented Understudy for Gisting Evaluation–Longest Common Subsequence] and BERTScore [Bidirectional Encoder Representations from Transformers Score]) and a qualitative human assessment across 5 domains, including correctness and patient-centeredness. Figure 1 provides an overview of the study workflow.

### First Step

#### Patient and Public Involvement

Two PPIE groups were recruited from Devon, a southwest coastal region in the United Kingdom. Eligible participants included adult patients (aged 18 y or older) with capacity to provide informed consent and engage in PPIE discussions, as well as clinicians who regularly conducted face-to-face clinical appointments. Patients were excluded if they lacked decision-making capacity or could not meaningfully participate. Clinicians were excluded if they did not routinely provide direct patient care, meaning those with continuous contact with patients in the outpatient clinic.

Semistructured interviews for the PPIEs were led by KG, who developed and iteratively improved and simplified the interview questions with input from experts in patient-centeredness. These questions explored the importance of capturing (1) personal and lifestyle information, (2) support systems and beliefs, (3) health goals and challenges, (4) emotional and mental health, (5) sources of meaning, and for clinicians, an additional question about how they preferred to receive and prioritize the information discussed from an appointment. These domains were derived and synthesized from integrative models of patient-centered care and from expert-validated frameworks that prioritize dimensions, such as the unique person, patient involvement, provision of information, patient-clinician communication, and empowerment [30,31]. These questions were implemented so that patients discussed what personal and contextual information should be captured in clinical summaries, while clinicians reflected on how such information could be structured for clinical use.

#### Data Analysis

Interview notes were handwritten during or shortly after sessions and thematically coded using the 5 patient-centered domains, along with an additional category for structural preferences. Data were stratified by participant age groups (young [18‐30 y], middle-aged [31‐60 y], and older [61‐91 y]) and role (patient or clinicians) and analyzed through iterative thematic analysis to identify common and divergent priorities. Relevant quotes were categorized and introduced into each subheading. The number of interviews was determined by the relevance and depth of contributions rather than by strict saturation thresholds, consistent with PPIE research guidelines [28,29]. Because the objective was to inform the development of the patient-centered summarization framework rather than exhaustively identify themes, interviews continued until additional data were unlikely to meaningfully alter the structure or domains of the framework, consistent with the concept of information power [32]. Data collection occurred within 1 month and depended on the availability of general practice sites able to support participation.

### Second Step

#### Annotation Guideline and Gold Standard PCS

The results from the first step were used to develop the annotation guideline, which incorporated input from both the clinicians’ PPIE to guide the desired format and the patients’ PPIE to provide illustrative quotes and categories within each key area, as illustrated in Multimedia Appendix 1.

A team of 8 clinician annotators applied the guideline after a calibration phase to ensure shared understanding. In this phase, pairs worked on the same transcript, identifying background topics, issues, the plan, and patient-centered elements, and generating summaries until their outputs showed similar structure and complete inclusion of PCS elements. Disagreements were resolved through discussion.

Annotators generated 88 PCS from nonscripted patient-clinician consultations obtained from a SDM clinical trial in atrial fibrillation [33]. A total of 922 encounters were originally recorded. Scripted encounters from the intervention arm were excluded because clinicians followed the decision aid step by step, limiting exploration of other aspects of patients’ lives. Encounters were also excluded when essential components of the consultation structure, such as a clear discussion and agreement on a management plan, were missing. After applying these criteria, the team purposively selected 88 eligible encounters to ensure diversity in patient and clinician characteristics as well as in communication styles. Original clinical notes were not used as a comparator, as they are clinician-oriented and focus primarily on biomedical aspects. In contrast, transcriptions captured the full dialogue, including patients’ perspectives and contextual details, providing a comprehensive and ethically accessible source for developing PCS.

#### Data Analysis

We quantified interannotator agreement using percent agreement and Gwet AC1, consistent with previous clinical annotation studies, which adjust for chance agreement while being less sensitive to label imbalance and the κ paradox [34,35]. For each consultation, 2 annotators independently generated summaries. Interrater agreement was calculated by coding the presence of information items and patient-centered elements using binary ratings (1=present, 0=absent). Percent agreement and Gwet AC1 were calculated across all coded elements. Final mean percent agreement was 92.6% with an overall mean Gwet AC1 of 0.91 (SD 0.12), and 92% and 0.88 for patient-centered elements, indicating excellent reliability.

### Third Step

#### Prompt Generation

Out of 88 transcribed patient-clinician conversations, we selected 16 (18%) for prompt generation. The prompt Multimedia Appendix 2 was iteratively improved based on the annotation guideline, with instructions for generating each section of the clinical summary refined through comparison between AI-generated outputs and gold standard PCS.

Open-source models were selected because the study data included sensitive real-world clinical conversations, and ensuring full deidentification of personally identifiable information would have introduced additional privacy risks and administrative burdens. The use of closed-source or externally hosted LLMs is constrained by institutional data governance policies, as these systems require transmission of data to third-party servers. Although some institutions permit their use under specific agreements, such arrangements limit reproducibility and broader applicability across settings. Accordingly, proprietary models and certain recently released systems (eg, DeepSeek) were excluded under our institution’s strict information security and vendor risk management policies. To ensure compliance with data privacy requirements and maximize generalizability in real-world health care settings, all selected models supported local, offline inference, allowing full control over data handling and ensuring data security.

#### Data Analysis

Performance during the prompt refinement phase was assessed using a dual approach. First, we performed a qualitative alignment check against the gold standard summaries. Second, quantitative metrics, specifically ROUGE-L for lexical similarity and BERTScore for semantic alignment, were calculated to provide objective feedback for iterative prompt adjustments

### Fourth Step

#### Performance Assessment

The 5 selected open-source LLMs generated summaries from 72 transcribed patient-clinician consultations using zero-shot and few-shot (1‐3 examples) prompting. For all models, hyperparameters were standardized, with temperature fixed at 1 and the maximum number of new tokens set to 2048, as the gold standard PCS did not exceed this length. The study evaluated pretrained open-source models without additional training or fine-tuning. During inference, we used a batch size of 1 and greedy search as the decoding strategy and used a single NVIDIA L4 GPU with 24 GB of memory.

#### Data Analysis

Quantitative performance was evaluated against the gold standard PCS using ROUGE-L for lexical similarity and BERTScore for semantic alignment. ROUGE-L was calculated using the standard *F*_1_ formulation, which measures the longest common subsequence between the model output and the reference summary; scores range from 0 to 1, with higher values indicating greater overlap (eg, a ROUGE-L of 0.2 reflects relatively limited lexical similarity) [36]. BERTScore, also normalized between 0 and 1, was computed using contextual embeddings to capture semantic similarity; higher scores reflect closer alignment in meaning between system and reference summaries [37].

In the descriptive analysis, we first quantified the amount of patient-centered information present in the source transcripts. Each consultation was manually labeled according to our patient-centered framework, and the number of patient-centered statements per encounter was recorded. Across the 72 consultations, this number ranged from 0 to 16.

A qualitative assessment was performed using the framework proposed by Van Veen et al [38], which we adapted to evaluate summaries across 5 domains—correctness, completeness, conciseness, patient-centeredness, and fluency. To assess patient-centeredness, we identified which elements of the PCS framework were present in each summary using the labeled transcripts and quantified the amount of patient-centered content included. Furthermore, 5 pilot summaries were then rated and discussed to calibrate the approach, and afterward 2 reviewers (MLJ and AGC) independently rated all summaries. This assessment was conducted in a blinded strategy, as evaluators did not know whether the summaries had been generated by the model or by human experts. For this purpose, summaries were anonymized, randomized, and labeled only as summary A and summary B. Each domain was scored on a scale from –5 to +5, where negative values favored the gold standard PCS, positive values favored the AI-generated summaries, and 0 indicated no difference; directionality was assigned after unblinding to identify which summary corresponded to each source. Scoring was guided by predefined rules; for correctness, we reviewed whether the information in each summary was factually accurate according to the transcript. After unblinding, instances where the model introduced information that was not present in the source, such as inventing medications or clinical details without supporting context, were classified as hallucination and rated –5 if more than 2 instances were present or −3 if 1 instance was identified. For patient-centeredness, we compared summaries with tagged transcripts and assigned 0 if both contained a similar amount of patient-centered content, –3 or +3 if 1 included less than half of the tagged content, and –5 or +5 if 1 included more than half, depending on which summary achieved this.

To assess interrater reliability between the 2 evaluators (MLJ and AGC), we conducted a pilot evaluation using 3 videos, each rated across 5 parameters on an ordinal scale ranging from −5 to +5. Interrater agreement was quantified using the quadratic-weighted Cohen κ coefficient, which accounts for the ordinal nature of the data by assigning smaller penalties to minor rating differences. The resulting κ value was 0.84, indicating excellent agreement [39].

### Ethical Considerations

The study was conducted in accordance with the International Conference on Harmonization Good Clinical Practice guidelines. The study protocol was approved by the Mayo Clinic Institutional Review Board (IRB#25‐007747) and included approval for the secondary analysis of data derived from the atrial fibrillation clinical trial. Written informed consent was obtained from all participants involved in both the PPIE groups and the original clinical trial.

Regarding participant compensation, patients did not receive direct financial remuneration for their participation in the PPIE groups or the original clinical trial. However, no additional travel, logistical, or incidental expenses were incurred outside of their routine clinical care. No participant suffered financial detriment. Clinicians were compensated for their time according to a fixed hourly institutional rate to offset the direct loss of billable clinical hours required for participation.

## Results

### Patient-Centered Summarization Framework

Through the PPIE participants, including 8 clinicians and 10 patients representing diverse age groups (Table 1), we identified essential themes to guide patient-centered summarization according to patients (Table 2 and Multimedia Appendix 3). Their insights informed the development of a structured framework with the following five domains: (1) lifestyle and daily routines, (2) support systems and beliefs, (3) health goals and challenges, (4) emotional and mental health, and (5) sources of meaning.

**Table 1.** Demographics of PPIE^a^ participants. The table summarizes the composition of the PPIE groups, including clinicians and patients, stratified by age group, sex distribution, and percentage of the total sample.

Group	Age range (y)	Sex, n (% of group)	Total, n (% of all)
General practitioners	—^b^	—	7 (38.9)
District nurses	—	—	1 (5.5)
Young patients	18‐30	Male: 2 (66.7) Female: 1 (33.3)	3 (16.7)
Middle-aged patients	31‐60	Male: 1 (33.3) Female: 2 (66.7)	3 (16.7)
Older patients	61‐91	Male: 3 (75) Female: 1 (25)	4 (22)
Total patients	—	Male: 6 (60) Female: 4 (40)	10 (55.5)

a PPIE: patient and public involvement and engagement.

b Not available.

**Table 2.** Themes identified using the patient-centered framework. Themes identified from the patient and public involvement and engagement interviews, differentiated by older, middle-aged, and younger patients. The table summarizes perspectives across 5 domains: lifestyle and daily routines, support systems and access, events and life stressors, care preferences, and sources of meaning or value.

Category	Older patients (61‐91 y)	Middle-aged patients (31‐60 y)	Younger patients (18‐30 y)
Lifestyle and daily routines	Active in managing health (eg, monitoring blood pressure and volunteering) [Patient A, C]	Shared limited lifestyle details (eg, physical job and retired builder) [Patients H, I]	Limited lifestyle information; concerns about chronic pain [Patient E]
Support systems and access	Reliance on friends for transport; more care needed from loved ones [Patient C]	Family support during crises; challenges with relatives’ health [Patients H, I]	Emotional support from family and friends; support sometimes is hard because close friends are often busy [Patient D]
Events and life stressors	Bereavement and chronic illness impact	Bereavement and family illness noted but not shared often	Extrinsic factors like trauma, relationships, and work stress
Care preferences	Preference for continuity and face-to-face care; avoid technology for engagement [Patient J]	Limited specific preferences	Specific preferences based on condition (eg, medication avoidance) [Patients D]
Sources of meaning or value	Community contributions and self-management	Less emphasis on sources of meaning unless stressed	Importance of work and family as motivators

### Lifestyle and Daily Routines

Older patients emphasized the importance of documenting daily routines as indicators of independence and health engagement. For example, they shared efforts to manage blood pressure, engage in volunteering, or maintain physical activity despite mobility challenges. In contrast, middle-aged and younger patients provided fewer details unless health issues clearly affected their routines. When symptoms disrupt daily functioning, such as chronic pain or fatigue, younger patients consider lifestyle information more relevant to record.

### Support Systems and Beliefs

Across age groups, patients valued recording the presence and quality of social support, especially during health crises. Older patients described logistical challenges, such as transportation and reliance on friends, while younger and middle-aged patients referred to emotional support from family as relevant, although sometimes inadequate. These narratives underscored how both practical and emotional support systems influenced access to care and coping strategies.

### Health Goals and Challenges

Patients of all ages described life stressors affecting their physical and emotional well-being. Younger participants highlighted the impact of trauma, relationship conflict, caregiving roles, and system-level stressors (eg, child protection fears). Older patients emphasized bereavement or caregiving burdens as key stressors. Some participants expressed a desire for clinicians to recognize how these challenges affected motivation, resilience, and engagement with care.

### Emotional and Mental Health

Preferences for emotional support and mental health disclosure varied. Some younger patients shared significant psychological distress, including suicidality or depression linked to life events. Others wanted clinicians to recognize emotional triggers but preferred sensitive handling. Older patients voiced frustration when distress was dismissed, especially when their usual coping mechanisms failed. The findings revealed that acknowledging emotional states is crucial to understanding health behavior and care needs.

### Sources of Meaning and Personal Values

Older patients identify meaning through community engagement, independence, and contribution, with some explicitly linking these to care preferences, such as avoiding residential care or choosing not to be resuscitated. Middle-aged and younger participants are less often named sources of meaning, unless those values (eg, work and parenting) were under threat. When identified, these sources of meaning shaped their outlook, motivation, and desire for personalized care planning.

Clinicians also expressed strong support for integrating person-centered information into clinical summaries, emphasizing the importance of understanding patients’ functional baselines, psychosocial context, and emotional well-being. Many described their role not only as medical providers but also as informal therapists or coaches, often encountering complex cases rooted in bereavement, loneliness, trauma, or caregiving stress. Several clinicians advocated for routinely documenting elements such as support networks, mental health history, and care preferences (eg, avoiding invasive treatments), as well as patients’ ideas, concerns, and expectations.

At the same time, clinicians noted practical considerations for summarization and implementation. Most preferred brief, structured summaries using bullet points, incorporating psychosocial data when relevant. Suggestions for system integration included embedding person-centered content into existing EHR tabs or flagging key insights with visual icons. Some clinicians proposed advanced uses of AI to visualize longitudinal patterns of psychosocial and health-related events or to generate personalized follow-up letters that were humane and accessible. However, a few raised philosophical concerns about whether AI could meaningfully capture the relational and dynamic aspects of healing, particularly the transformative role of the patient-clinician interaction itself.

### Baseline Characteristics

A total of 72 transcribed patient-clinician consultations from the atrial fibrillation trial were analyzed, with general characteristics described in Table 3. The mean patient age was 70 (SD 11) years, and 44.4% (32/72) were female. Most consultations took place at Mayo Clinic Rochester (25/72, 34.7%). While all encounters were related to atrial fibrillation, they occurred in different clinical settings, most commonly cardiology appointments (40/72, 55.6%), followed by thrombophilia clinic (9/72, 12.5%). The most prevalent comorbidity was a history of hypertension, reported in 87.5% (63/72) of participants.

**Table 3.** Demographic and clinical characteristics of patients participating in the atrial fibrillation conversations. Characteristics are reported for the 72 patient-clinician encounters included in the analysis, including demographic variables, clinical context, appointment characteristics, and care settings.

General characteristics	Total (N=72)
Age (y), mean (SD)	70 (11)
Sex, n (%)	
Female	32 (44.4)
Male	40 (55.6)
BMI (kg/m^2^), mean (SD)	33.0 (7.71)
Location, n (%)	
Mayo Clinic, Rochester, Minnesota	25 (34.7)
Park Nicollet, Minnesota	19 (26.4)
Hennepin County Medical Center	12 (16.7)
Alabama	4 (5.6)
Mississippi	12 (16.7)
Appointment length (min), mean (SD)	33.8 (17.5)
Appointment type, n (%)	
Emergency department	1 (1.4)
Primary care or family medicine	4 (5.6)
Inpatient	4 (5.6)
Cardiology	40 (55.6)
Thrombophilia clinic	9 (12.5)
Parkside ambulatory	2 (2.8)
Brooklyn Center Ambulatory center	7 (9.7)
Anticoagulation Clinic	3 (4.2)
Other	2 (2.8)
History of	
Hypertension, n (%)	63 (87.5)
Congestive heart failure, n (%)	13 (18.1)
Stroke, n (%)	12 (16.7)
Stroke type, n (%)	
TIA^a^	3 (25)
Ischemic stroke	9 (75)
Vascular disease, n (%)	21 (29.2)
Diabetes, n (%)	30 (41.7)
Renal disease, n (%)	10 (13.9)
Liver disease, n (%)	4 (5.6)
MI^b^, n (%)	5 (6.9)
PAD^c^, n (%)	2 (2.8)

a TIA: transient ischemic attack.

b MI: myocardial infarction.

c PAD: peripheral artery disease.

### Quantitative Evaluation

In the quantitative evaluation, the best performance in the zero-shot setting was achieved by Mistral-8B on ROUGE-L (0.189) and Llama-3.1-8B on BERTScore (0.673). As few-shot attempts were introduced, performance improved across all models. With 1 example, Llama-3.1-8B outperformed all others, achieving a ROUGE-L of 0.201 and a BERTScore of 0.680. The best-performing model overall was Llama-3.1-8B with 3-shot prompting, which achieved a ROUGE-L of 0.206 and a BERTScore of 0.683.

### Qualitative Evaluation

Only 1 consultation had no patient-centered content, while among the remaining 71, 10 included a single domain and 61 incorporated multiple domains. Patient-centered topics spanned lifestyle and daily routines, support systems, life stressors, care preferences, and sources of meaning.

**Figure 2.**
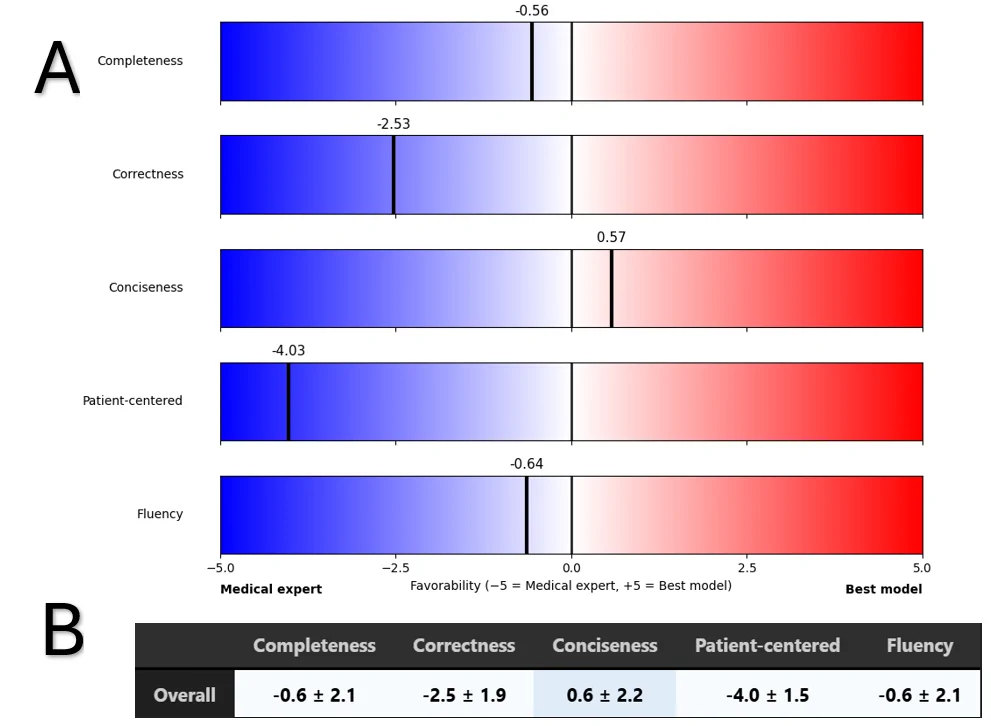
Qualitative comparison between artificial intelligence–generated summaries and gold standard patient-centered summaries across consultations (n=72). (A) Diverging bar plots show mean domain scores (range −5 to +5) for completeness, correctness, conciseness, patient-centeredness, and fluency; negative values favor the gold standard summaries and positive values favor the artificial intelligence–generated summaries. (B) Table summarizing mean±SD scores for each domain. Gold standard summaries showed higher correctness and patient-centeredness, while conciseness slightly favored the model.

The qualitative human evaluation compared the AI-generated summaries from the best-performing model against the gold standard PCS (Figure 2). The 2 approaches performed similarly in terms of completeness (mean –0.6, SD 2.1) and fluency (mean –0.6, SD 2.1). However, the gold standard PCS were rated significantly higher for correctness (mean –2.5, SD 1.9), as the model occasionally hallucinated procedures and medications not mentioned in the consultations. Conciseness slightly favored the model (mean 0.6, SD 2.2). The most substantial difference was in patient-centeredness, where gold standard PCS vastly outperformed the model (mean –4.0, SD 1.5), reflecting their superior ability to capture the nuanced details patients shared about their daily routines, support networks, and personal values. On average, transcripts contained (mean 3.6, SD 2.5) patient-centered domains, whereas AI-generated summaries captured only (mean 0.8, SD 0.9), and nearly half (35/72, 49%) contained none. Illustrative qualitative comparisons between gold standard and AI-generated PCS are provided in Multimedia Appendix 4.

## Discussion

### Principal Findings and Relevance

In this study, we developed a PCS framework informed by PPIE and evaluated the performance of open-source LLMs in capturing patient-centered elements in clinical summaries. Through PPIE, patients identified 5 key domains as essential to their care—lifestyle and daily routines, support systems, health goals and challenges, emotional and mental health, and sources of meaning and personal values. Clinicians similarly supported the inclusion of patient-centered information, highlighting the importance of understanding patients’ functional baselines and psychosocial context while emphasizing the value of brief, structured, and clinically accessible summaries. When applied to real-world atrial fibrillation consultations, patient-centered information was better captured in the gold standard PCS and was inconsistently present in AI-generated summaries, with a mean patient-centeredness rating of −4.0 (SD 1.5) on the −5 to +5 evaluation scale. Although commonly used summarization metrics, such as ROUGE-L and BERTScore, were consistently low across all AI-generated PCS, qualitative evaluation of the best AI-generated PCS with few-shot prompting were comparable with the gold standard PCS in conciseness, completeness, and fluency but were inferior in correctness (ie, hallucinations) and patient-centered information.

Standard clinical AI summarization research primarily targets clinician-oriented documentation, such as visit or discharge notes, emphasizing biomedical information extraction and documentation efficiency [13,18]. For example, recent studies generate structured clinical notes from patient-clinician conversations using LLMs and prioritize diagnoses, medications, and care plans for clinician use, while earlier approaches focused on compressing clinical records for professional documentation [40-42]. In contrast, our PCS framework explicitly incorporates patient-identified domains, positioning PCS as a methodologically distinct subtype of AI clinical summarization, rather than a conventional documentation-focused task.

Previous research across multiple health care settings demonstrates the value of patient-centered written summaries for supporting patient understanding and engagement. In outpatient care, patient-centered after-visit summaries have been associated with improved patient awareness and medication-related behaviors, although they frequently exhibit suboptimal design features, including high reading grade levels and poor usability [19,43]. Similarly, the introduction of patient-centered information in cancer consultation summaries has been linked to improved patient-centered communication and perceived quality of care, yet these summaries remain inconsistently implemented in practice [44,45]. In this context, creating a rapid, contextualized approach to create a framework to generate PCS might be impactful for patient care outcomes.

The domains identified in our framework align with previous work aiming to incorporate holistic patient perspectives into care documentation and planning [46,47]. For instance, in our PCS framework, older participants emphasized independence and daily functioning, reflecting well-documented age-specific stress perception and priorities. As highlighted by the American Psychological Association, declines in functional abilities increase reliance on environmental supports to maintain autonomy and quality of life, reinforcing older adults’ focus on daily routines and health-related function [48]. In contrast, younger participants in our study discussed emotional burdens and system-level stressors, including work, finances, and relationships, patterns consistent with evidence that younger adults report higher total daily stress and are more affected by interpersonal and occupational demands [49,50]. These age-specific differences between older adults (eg, independence) and younger adults (eg, occupational stress) imply that a “one-size-fits-all” AI prompting is insufficient. Future models should be able to adapt to each patient’s values and preferences to generate PCS.

In our study, clinicians expressed strong support for systematically capturing person-centered data. They emphasized that understanding patients’ functional baselines, emotional challenges, and preferences are central to care, according to the biopsychosocial model widely encouraged in primary care literature [23]. At the same time, clinicians highlighted practical constraints, advocating for summaries that are brief, have structure, and are clinically accessible. They suggested integrating person-centered content into discrete sections of the EHR (eg, icons or lifestyle tabs). These preferences align with previous studies where clinicians have expressed the need for EHR systems to facilitate intuitive, less burdensome documentation interfaces and to allow for both structured and narrative content, supporting both efficient data entry and the nuanced, person-centered aspects of care [51].

In our evaluation, gold standard PCS were notably stronger in patient-centeredness and correctness compared with AI-generated summaries, underscoring key limitations of current LLMs in clinical summarization. Despite modest gains with few-shot prompting, the models showed limited lexical similarity (ROUGE-L) and semantic alignment (BERTScore) with gold standard PCS. These findings contrast with previous work demonstrating strong LLM performance in biomedical clinical summarization and suggest that current models remain poorly equipped to capture personal, emotional, and value-based information [38]. This limitation likely reflects the predominance of biomedical and pathophysiological content in model training data [52]. The lack of dedicated datasets, annotation standards, and evaluation tools for PCS further contributes to this problem, making it difficult for models to capture the social, emotional, and functional information that matters most to patients and clinicians.

Hallucinations and factual inaccuracies remain a known limitation of LLMs and may introduce clinically unsafe information, such as incorrect medications, diagnoses, or management plans [53]. In this study, factual correctness was systematically evaluated by comparing the information from the generated summaries either by AI or humans to the information from the transcribed conversation. The addition of information not present on the transcriptions (ie, hallucinations) was more prevalent in the AI-generated PCS. Therefore, these summaries should not be used without clinician oversight.

### Limitations and Future Directions

Several limitations must be considered. First, the PCS framework was developed using PPIE conducted in the UK population and evaluated in patient-clinician consultations focused on a single clinical condition from the United States; this pragmatic decision was driven by the availability of a high-quality, IRB-approved dataset. While the core principles of patient-centeredness are universal, the specific expression and prioritization of patient concerns can be shaped by cultural and health system contexts. For instance, patients within the United Kingdom’s National Health Service may emphasize different priorities than navigating the US insurance system, where financial considerations can be a more prominent part of the illness experience [54]. As such, while our framework provides a robust foundational model, adaptation and validation across diverse health care systems are needed.

Second, because interviews were designed to inform framework development rather than achieve thematic saturation, the sample size and recruitment timeframe may have limited the breadth of perspectives captured. A larger or more diverse PPIE sample could identify additional priorities that might further refine the framework.

Third, while this study evaluated 5 widely used open-source LLMs to ensure transparency and reproducibility, the selection of models was also shaped by institutional data constraints. Because the study involved sensitive real-world patient-clinician conversations, the use of closed-source or externally hosted models requiring data transmission to third-party servers was not permitted. In clinical practice, strict data governance often precludes the transmission of sensitive patient-clinician audio transcripts to third-party cloud providers [55]. By benchmarking open-source models that can be deployed on-premise, we establish a baseline for secure, HIPAA (Health Insurance Portability and Accountability Act)-compliant AI summarization, a prerequisite for real-world adoption.

Fourth, commonly used quantitative metrics such as ROUGE and BERTScore [36,37] have known limitations, as they emphasize lexical and semantic overlap and do not fully capture patient-centeredness. This limitation motivated the use of domain-specific categories and structured qualitative human evaluation by using our patient-centered framework, which shows that AI-generated PCS can be still useful regardless of their automated metrics scores.

Finally, in this initial evaluation, we did not use retrieval-augmented generation, as our primary focus was on the PPIE and qualitative components. Future AI systems should be able to leverage recorded clinical conversations to derive each patient’s values, preferences, and wishes, and incorporate this information into summarization AI models to truly generate individualized PCS [56]. The deployment of such systems should be evaluated in pragmatic clinical trials to assess their impact on care outcomes and should be guided and overseen by health care professionals to avoid potential risks such as hallucinations. In future studies, we will compare fine-tuning and retrieval-augmented generation approaches and explore the development of our own model using a larger dataset.

### Conclusion

This study shows that producing truly patient-centered clinical summaries remains a major challenge for current open-source LLMs. Using a mixed methods framework grounded in patient and clinician input, we identified the key domains of a PCS. The tested models failed to capture these dimensions, particularly factual correctness and nuanced patient values, with ROUGE-L and BERTScore results well below human benchmarks. Our work provides a validated framework, a reference standard for future research, and a pathway toward patient-centered AI summarization. Future work should focus on developing AI summarization models that can include individualized patient-centered elements in clinically meaningful summaries and evaluate its impact on clinical and care outcomes in clinical trials.

## Supplementary material

10.2196/87061 Multimedia Appendix 1 Annotation guideline.

10.2196/87061 Multimedia Appendix 2 Prompt.

10.2196/87061 Multimedia Appendix 3 Illustrative examples of how patients in different age groups described their experiences across thematic categories (Lifestyle and daily routines, support systems and access, events and life stressors, care preferences, and sources of meaning or value), corresponding to the summary presented in Table 2.

10.2196/87061 Multimedia Appendix 4 Comparison of gold standard patient-centered summaries (PCS) and model-generated patient-centered summaries. Representative excerpts (“chunks”) are presented side by side to illustrate qualitative differences in clinical content, structure, and inclusion of protocol-defined patient-centered elements. Two illustrative cases (Example #1 and Example #2) are shown. To protect patient privacy, full summaries are not displayed. Selected excerpts are organized by summary sections (Background, Issues, Plan and PCS domains). Domain-specific qualitative evaluation scores (completeness, correctness, conciseness, fluency, and patient-centeredness) are reported after each example.

## Abbreviations

AI: artificial intelligence
BERTScore: Bidirectional Encoder Representations from Transformers Score
EHR: electronic health record
IRB: Institutional Review Board
LLM: large language model
PCS: patient-centered summary
PPIE: patient and public involvement and engagement
ROUGE-L: Recall-Oriented Understudy for Gisting Evaluation–Longest Common Subsequence
SDM: shared decision-making
