# Supplementary material for: Patient-Centered Summarization Framework for AI Clinical Summarization: Mixed Methods Study
Source: J Med Internet Res. 2026 Sep 3;28:e87061. doi: 10.2196/87061 (PMC13541227; doi:10.2196/87061)
Supplement: Supplementary file 1 [file jmir-v28-e87061-s001.docx]

*Supplementary material S1*

*Annotation Guideline*

*Instructions*

*Step #1:*

- Elaborate the patient-centered (PC) summaries first. [Read about patient-centered information – see word documents]
- Generate summary by **only** watching the video – do not use/read the transcripts while the video is playing. You can read the transcripts, but you should stop the video when you do so.
- Do not forget to highlight in yellow the PC information on the PC summaries. This will help you in the second step and it is there to help all of us (the researchers) where the PC information is.
- Outline of the PC summary

*Background [bg1]:* Past medical history, including an ongoing medical condition such as Atrial fibrillation, and age. For instance, patient goes to a follow-up appointment of his AFib medication; this will be considered both as background and issues. Do this in chronological order based on how it is mentioned on the video.

*EVERY medical condition* that has any type of information (e.g., medication, PC information, follow-up info, doctor who is following the condition) should go in one bullet

*All medical conditions* that are just listed without any additional information should go all of them in ONE bullet point.

*EVERY Medication* that is related to a medical condition should be in the bullet that corresponds to the medication.

*All medications* that are just listed and not related to medical conditions spoken during the consultation should be all lumped together (grouped into one bullet).

*You can have BG with and without PC information*

- Background with PC information
- Background without PC information

*Issues [issue1]:* In chronological order.

- Issues can have PC information
- Issues without PC information

*Plan [plan1]:*  Write the plan based on the orders of the issues.

*Step #2:*

*PLEASE BE AS PRECISE AS POSSIBLE WHEN TAGGING YOUR SENTENCES.* Do not include unnecessary information.

- Tag the parts of the transcripts that helped you elaborate the PC summary
- You will do this by reading your patient-centred summary and identifying the sentences from the transcript the helped you elaborate this summary.
- The minimum unit for tagging is a sentence. Sentences are defined as those that end with a period “.” or exclamation marks (“?”, “!”).

*A tag can never end within a sentence. It should end at the end of the sentence (read the definition of sentence on the previous point, please)*

- You need to tag these sentences based on the following nomenclature.
- Background -> Tags -> no order
- beginning -> [bg1]
- Final -> [/bg1]
- Issues - order
- Organise issues by number. Tags
- [issue1]
- [/issue1]
- Plan - order
- [plan1]
- [/plan1]
