# Supplementary material for: Patient-Centered Summarization Framework for AI Clinical Summarization: Mixed Methods Study
Source: J Med Internet Res. 2026 Sep 3;28:e87061. doi: 10.2196/87061 (PMC13541227; doi:10.2196/87061)
Supplement: Supplementary file 3 [file jmir-v28-e87061-s003.docx]

*Supplementary material S3*

Supplementary Material S3. Illustrative examples of how patients in different age groups described their experiences across thematic categories (Lifestyle & Daily Routines, Support Systems & Access, Events & Life Stressors, Care Preferences, and Sources of Meaning/Value), corresponding to the summary presented in Table 2.

*Lifestyle & Daily Routines:*

- *Patient A* (older) monitored blood pressure and supported pharmacy routines.
- *Patient C* (older/middle-aged) emphasized healthy lifestyle and volunteering.
- *Patient H* (middle-aged) described work as physically demanding; *Patient I* (middle-aged) retired from building work.
- *Patients F and G* (younger) did not share lifestyle details.
- *Patient E* (younger) reported chronic pain affecting daily life.

*Support Systems & Access:*

- *Patient J* (older) Issues accessing General practitioners.
- *Patient C* (older) More care needed for/from loved ones.
- *Patient H* (middle-aged) mentioned mother’s health issues; *Patient I* (middle-aged) reported dealing with family health problems.
- Patient D (younger) described his close friends as often being busy; Patient E (younger) noted that his wife provided limited support, not the needed kind; Patient G (younger) identified his mother as a key source of support.

*Events & Life Stressors:*

- *Patient B* (older) had suicidal risk due to mobility.
- *Patient H* (middle-aged) affected by mother’s Chronic Obstructive Pulmonary Disease; *Patient I* by aunt’s dementia.
- *Patient F* (younger) experienced past traumas and relationship difficulties; *Patient D* reported fear of social services.

*Care Preferences:*

- *Patient J* (older) preferred face-to-face care and avoided technology for engagement.
- *Patients C, F, G, H, I* (middle-aged) expressed no specific preferences.
- Patient D (younger) avoided Selective serotonin reuptake inhibitor and required encouragement; Patient E emphasized the need for clear medical history summaries.

*Sources of Meaning/Value:*

- Patient A (older) found meaning in the community; Patient C in mental health.
- *Patient H* (middle-aged) emphasized community connectedness.
- Patient D (younger) described children as motivators; Patient E cited a small business as a source of distraction and meaning.
