# Supplementary material for: Patient-Centered Summarization Framework for AI Clinical Summarization: Mixed Methods Study
Source: J Med Internet Res. 2026 Sep 3;28:e87061. doi: 10.2196/87061 (PMC13541227; doi:10.2196/87061)
Supplement: Supplementary file 4 [file jmir-v28-e87061-s004.docx]

**Supplementary material S4**

| ***Gold-Standard Patient-Centered Summaries*** | | ***AI-Generated Patient-Centered Summaries*** |
| --- | --- | --- |
| ***Example #1*** | | |
| ***Background*** | | |
| "Atrial fibrillation, on warfarin, dofetilide and propafenone. Rheumatoid arthritis in Orencia. Dyastolic dysfunction, on furosemide. Diabetes in the setting of prednisone use. Sleep apnea on CPAP. Hypertension" | | "... history of hypertension, congestive heart failure, stroke, valvular disease, and diabetes.  - Current medications: warfarin, furosemide, Rythmol, Tikosyn, and CPAP" |
| ***Issues*** | | |
| “Atrial fibrillation: has been experiencing intermittent episodes of arrithmia. She uses dofetilide and propafenone. She is anticoagulated on warfarin 5 mg Monday, Wednesday, Friday and 2.5 mg the rest of the days. She is hesitant to try newer oral anticoagulants, citing fear and a lack of understanding about them. Her sister passed away while on one of these medications. Her risk of stroke was evaluated in lieu of the risk reduction and potential bleeding with anticoagulation. Based on her risk profile, she benefits from continued anticoagulation. The newer alternative agents were discussed, including eliquis, xarelto, and pradaxa. As compared to warfarin, the newer agents have fixed dosing, and do not have diet interactions, but have a higher cost and do not have reversal agents in case of bleeding. Given that warfarin appears to be working for her, she would prefer to continue with the same regimen at this time.” | | “Atrial fibrillation: The patient has a history of atrial fibrillation, with 3 episodes out of 15 visits since 2012. His heart function is strong, but he has diastolic dysfunction, which may require Lasix.\n- Rheumatoid arthritis: The patient has been taking Orencia for 3 months, which has finally started to show improvement in his symptoms. However, he is experiencing frequent urination as a side effect.”  “Warfarin: The patient is currently taking warfarin but is considering switching to a novel anticoagulant due to concerns about diet restrictions and monitoring.”  “Rythmol: The patient is taking Rythmol as needed to control his atrial fibrillation, which is affecting his sleep.” |
| ***Plan*** | | |
| “Continue anticoagulation with warfarin"  “Return in 6 months” | | “The doctor will send a prescription for Eliquis to the pharmacy to check the price and discuss the cost"  “The patient will consider the cost and make a decision about whether to switch to Eliquis or continue with Warfarin”.  “The doctor will continue to monitor the patient's INR levels and adjust their Warfarin dosage” |
| ***Patient Centered Information*** | | |
| “She is hesitant to try newer oral anticoagulants, citing fear and a lack of understanding about them.”  “Given that warfarin appears to be working for her, she would prefer to continue with the same regimen at this time.” | *No patient-centered information was included.* | |
| ***Scores of qualitative assessments***   - ***Completeness (-5):*** We highlighted in green the elements that were absent from the model generated summaries. - ***Correctness (–5):*** We highlighted in yellow the hallucinations identified within the model-generated summaries. - ***Conciseness (3):*** Although not directly illustrated in the example, when the model retained all relevant information, it presented the same content in a shorter format. - ***Fluency (0):*** Both summaries demonstrated similar readability, and no differences in fluency were observed. - ***Patient centeredness (–5):*** We highlighted in blue the patient centered information that was not included by the model. | | |
| ***Example #2*** | | |
| ***Background*** | | |
| 1- Atrial fibrillation diagnosed in August of 2014: currently on Warfarin.  2- Diabetes  3- Hypertension  4- Left elbow surgery on June 20^th^.  5- Previously bilateral hip replacement” | | “- Atrial Fibrillation (AF) diagnosis in August 2014  - On Warfarin since then  - Previous experience with Xarelto, which was discontinued due to the "donut hole" |
| ***Issues*** | | |
| “Atrial fibrillation: The patient's one-year stroke risk was estimated at 4%, potentially reduced by two-thirds with anticoagulation use, and a discussion took place regarding the benefits of this therapy. The risks of bleeding were reviewed, along with a detailed comparison of available medication options, highlighting their pros and cons. Key factors, such as the need for regular monitoring, reversibility of the medication, and associated costs, were also addressed.  The patient expressed particular concern about the cost differences and the impact of insurance coverage. She mentioned that was previously taking Xarelto but had to switch to Warfarin after reaching the "donut hole" due to cost issues, which caused significant frustration. She expressed a desire to avoid a similar situation in the future and showed interest in Eliquis as a potential option, depending on insurance coverage and affordability.” | | “The patient is weighing the convenience of switching to a new anticoagulant (Eliquis) against the cost, which is a significant factor in their decision-making process. The patient is concerned about the "donut hole" issue, which can result in high out-of-pocket costs for medications. The patient is interested in learning more about the cost of Eliquis and how it compares to Warfarin. The patient is considering switching to Eliquis due to its potential safety and convenience benefits, but is hesitant due to the cost” |
| ***Plan*** | | |
| “Consider switching to Eliquis depending on insurance coverage” | | “- The doctor will send a prescription for Eliquis to the pharmacy to check the price and discuss the cost, including the potential for the "donut hole"” |
| ***Patient Centered Information*** | | |
| “The patient expressed particular concern about the cost differences and the impact of insurance coverage. She mentioned that was previously taking Xarelto but had to switch to Warfarin after reaching the "donut hole" due to cost issues, which caused significant frustration. She expressed a desire to avoid a similar situation in the future and showed interest in Eliquis as a potential option, depending on insurance coverage and affordability.” | | “The patient is weighing the convenience of switching to a new anticoagulant (Eliquis) against the cost, which is a significant factor in their decision-making process. The patient is concerned about the "donut hole" issue, which can result in high out-of-pocket costs for medications. The patient is interested in learning more about the cost of Eliquis and how it compares to Warfarin. The patient is considering switching to Eliquis due to its potential safety and convenience benefits, but is hesitant due to the cost” |
| ***Scores of qualitative assessments***   - ***Completeness (-3):*** We highlighted in green the elements that were absent from the model generated summaries. - ***Correctness (0):*** We observed no hallucinations in the model generated summary, as both versions presented equivalent information. - ***Conciseness (3):*** Although not directly illustrated in the example, when the model retained all relevant information, it presented the same content in a shorter format. - ***Fluency (0):*** Both summaries demonstrated similar readability, and no differences in fluency were observed. - ***Patient centeredness (0):*** We highlighted in blue the patient centered information included in both summaries. | | |

***Supplementary Table 4.*** *Comparison of gold-standard* patient-centered summaries (PCS) and model-generated patient-centered summaries. Representative excerpts (“chunks”) *are presented side by side to illustrate* qualitative *differences in clinical content, structure, and inclusion of* protocol-defined *patient-centered elements. Two illustrative cases (Example #1 and Example #2) are shown.* To protect patient privacy, full summaries are not *displayed.* Selected excerpts are *organized* by summary sections (Background, Issues, Plan and PCS domains). *Domain-specific qualitative evaluation scores (completeness, correctness, conciseness, fluency, and patient-centeredness) are reported after each example. AI: Artificial Intelligence*
